# Correlation Between Blood Glucose Variability and Early Therapeutic Effects After Intravenous Thrombolysis With Alteplase and Levels of Serum Inflammatory Factors in Patients With Acute Ischemic Stroke

**DOI:** 10.3389/fneur.2022.806013

**Published:** 2022-02-22

**Authors:** Yun Cai, Hongtao Zhang, Qiang Li, Peilan Zhang

**Affiliations:** ^1^Clinical College of Neurology, Neurosurgery and Neurorehabilitation, Tianjin Medical University, Tianjin, China; ^2^Department of Neurology, Affiliated Hospital of Hebei University, Baoding, China; ^3^Department of Neurology, Tianjin Huanhu Hospital, Tianjin, China

**Keywords:** acute ischemic stroke, alteplase, thrombolytic therapy, blood glucose variability, inflammatory factors

## Abstract

**Objective:**

To investigate the effects of blood glucose variability on early therapeutic effects after intravenous thrombolysis with alteplase and the levels of serum inflammatory factors in patients with acute ischemic stroke (AIS).

**Methods:**

We enrolled AIS patients who received intravenous thrombolysis within 4.5 h of the onset of symptoms. Clinical data, including the National Institutes of Health Stroke Scale (NIHSS), glycosylated hemoglobin, mean blood glucose, standard deviation of blood glucose, mean amplitude of glycemic excursion, mean variation coefficient of blood glucose, interleukin-6 (IL-6), active matrix metalloproteinase-9 (MMP-9), tumor necrosis factor α (TNF-α), and hypersensitive C-reactive protein (hs-CRP) levels, were compared between a group who showed improvement (the improvement group) and a group who did not show improvement (the non-improvement group). Relevant factors for early neurological improvement after thrombolysis with alteplase were analyzed by using multivariate logistic regression models. A Pearson linear correlation analysis was also performed on blood glucose variation and inflammatory factor levels within the two groups.

**Results:**

A total of 146 patients were included, 63 of which had early symptom improvement (43.15%). The diabetes ratio, atrial fibrillation ratio, baseline NHISS score, random blood glucose at admission, and glycosylated hemoglobin of patients in the improvement group were significantly lower than those in the non-improvement group (*P* < 0.05 in all cases). The mean blood glucose, standard deviation of blood glucose, mean amplitude of glycemic excursion, and mean blood glucose variation coefficients of patients in the improvement group were significantly lower than those in the non-improvement group (*P* < 0.05). Serum inflammatory factor levels, including IL-6, MMP-9, TNF-α and hs-CRP, were significantly lower in patients in the improvement group compared to patients in the non-improvement group (*P* < 0.05). Multivariate logistic regression analysis showed that baseline NIHSS scores (OR = 1.28, 95% CI = 1.05–1.62, *P* = 0.02), glycosylated hemoglobin scores (OR = 2.57, 95% CI = 1.78–3.98, *P* = 0.0005), diabetes (OR = 13.10, 95% CI = 1.63~131.45, *P* = 0.021), the mean amplitude of glycemic excursion (OR = 2.98, 95% CI = 1.92–5.00, *P* < 0.0001), and the mean variation coefficient of blood glucose (OR = 1.40, 95% CI = 1.26–1.60, *P* = 0.0078) were significantly correlated with early symptom improvement after thrombolysis. Pearson linear correlation analysis showed that the standard deviation of blood glucose, mean amplitude of glycemic excursion, and the mean variation coefficient of blood glucose were significantly positively correlated with IL-6, MMP-9, TNF-α and hs-CRP levels (*P* < 0.01).

**Conclusions:**

Blood glucose variability is correlated with early neurological improvement after intravenous thrombolysis with alteplase in AIS patients. With the increase of blood glucose fluctuation range, the inflammatory response is enhanced, which affects the prognosis of patients.

Stroke is the disease with the highest incidence, disability, and mortality in China, with ischemic stroke accounting for 70% of disease burden. At present, the most effective treatment is intravenous thrombolytic therapy with recombinant tissue plasminogen activators (rt-PA) at early stages of the disease (<4.5 h) (1). Early improvement in neurological function after thrombolytic therapy is a marker of vascular recanalization and is independently predictive of good prognoses at 3 months. However, the efficacy of intravenous thrombolytic therapy is not always satisfactory. This may be due to many factors. Blood glucose and blood glucose variability are important risk factors for cardiovascular and cerebrovascular diseases, and blood glucose variation can induce the occurrence or aggravation of inflammatory responses. Recent studies have confirmed that blood glucose variability is an important prognostic factor that can independently predict death in critically ill patients. However, there is still no consensus about the influence of blood glucose variability on the effects of early thrombolytic therapy and inflammatory responses in AIS patients. Here, we studied AIS patients that were treated with intravenous thrombolytic therapy with alteplase. While strictly controlling for blood glucose levels and reducing the incidence of hypoglycemia, we evaluated the correlation between blood glucose variation levels, the early treatment effects of intravenous thrombolytic therapy with alteplase, and levels of serum inflammatory factors. We hope to guide effective blood glucose-based interventions, and to improve the therapeutic effects of intravenous thrombolysis in patients experiencing AIS.

## Patients and Method

### Patients

We included a total of 154 AIS patients who received intravenous thrombolysis with alteplase within 4.5 h of onset between August 2020 and June 2021. Among these patients, 8 cases were treated intravenously using bridging after intravenous thrombolysis, with a total of 146 cases eventually included in the final analysis.

#### Inclusion Criteria

(1) Age > 18. (2) Illness conformed to the “Guidelines for diagnosis and treatment of acute ischemic stroke 2018 in China”. (3) Stroke symptom duration was ≥30 min, National Institutes of Health Stroke Scale (NIHSS) score between 4 and 25 points, with no significant improvement in symptoms before thrombolytic therapy. (4) The standard of intravenous thrombolysis was met within 4.5 h of onset. (5) The clinical data were detailed and complete. (6) Patients or their families agreed to the study and signed informed consent forms.

#### Exclusion Criteria

(1) Patients with a history of severe stroke and mRS scores >2 before onset. (2) Patients with a history of severe head trauma or stroke within the past 3 months. (3) Patients with intracranial hemorrhage. (4) Patients with active hemorrhage. (5) Patients with a history of seizures. (6) Patients with serious insufficiencies within important organs, including the heart, lungs, liver, kidneys, and others. (7) Patients with inflammatory conditions, including but not limited to connective tissue disease, vasculitis, infection, tumor and acute myocardial infarction. (8) Patients that had recently used anti-inflammatory drugs, including glucocorticoids, immunosuppressants, biologics and non-steroidal anti-inflammatory drugs (excluding low-dose aspirin and statins). (9)Patients with blood pressure higher than 185/110 mmHg (1 mmHg = 133.32 Pa) after antihypertensive therapy. (10) Patients with random blood glucose levels <2.7 mmol/L at admission. (11) Patients with oral anticoagulant therapy and INR >1.5. (12) Patients with platelets t <100× 10^9^/L. (13) Cases where the time of onset was unknown. (14) Cases were intravenous thrombolysis was followed by endovascular bridging therapy. (15) Cases with incomplete clinical data. (16)Telephone or outpatient follow-up could not be completed.

This study was approved by the ethics committee of Tianjin Huanhu Hospital.

### Methods

#### General Information Collection

Baseline data, including gender, age, hypertension, diabetes, hyperlipidemia, coronary heart disease, atrial fibrillation, prior stroke/transient ischemic attack (TIA), smoking history, drinking history, onset to treatment (OTT), and NIHSS score before thrombolysis, were collected for all patients.

#### Treatment

Lyophilized alteplase powder (specification: 20 and 50 mg) produced by the German company, Boehringer Ingelheim, was used. Alteplase was dissolved in 100 ml normal saline at a total dose of 0.9 mg/kg (maximum dose was 90 mg), 10% of which was intravenously injected within 1 min, and the other 90% of which was intravenously injected within 1 h. Brain CT or MRI examinations were performed 24 h after thrombolysis. Patients without cerebral hemorrhage were treated with anti-platelet aggregation drugs and neuroprotection. Patients after cerebral infarctions were treated with insulin hypoglycemia when blood sugar was more than 10.0 mmol/L, and when blood sugar was below 3.3 mmol/L, 10–20% glucose was administered orally or by injection. Patients who were unable to eat orally were given a nasal fluid diet, with their blood glucose levels closely monitored.

#### Observation Indicators

**Figure 1:**
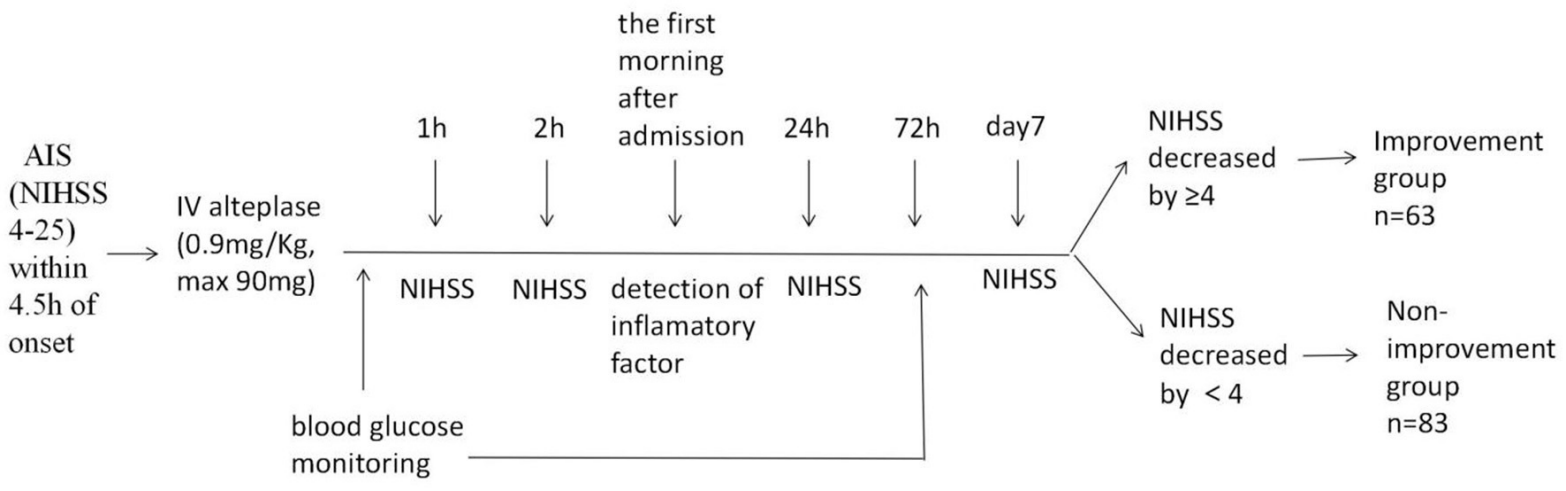
Study schema. AIS, acute ischemic stroke; NIHSS, National Institutes of Health Stroke Scale; IV, intravenous.

(1) Blood glucose monitoring: after admission, blood glucose was monitored every 4 h for 72 h using a Roche blood glucose meter. Then, the mean blood glucose (MBG), standard deviation of blood glucose (SDBG), mean amplitude of glycemic excursion (MAGE), and mean variation coefficient of blood glucose (MVCBG) were calculated. The last three indexes are used to measure the degree of variation in blood glucose levels. (2) Detection of inflammatory factors: in the morning following admission, 3 ml of fasting venous blood was extracted, and serum was separated. Interleukin-6 (IL-6), active matrix metalloproteinase-9 (MMP-9) and tumor necrosis factor α (TNF-α) were detected by enzyme-linked immunosorbent assay (ELISA), and the level of hypersensitive C-reactive protein (hs-CRP) were detected using scattering turbidimetry. (3) Efficacy evaluation: NIHSS scores were recorded at 1 h, 2 h, 24 h and 7 days after intravenous thrombolysis. Cases with NIHSS scores that decreased by ≥4 points 7 days after thrombolysis, or with neurological deficits that were completely resolved (i.e., NIHSS score = 0), were defined as having significant early improvement, as shown in Figure 1.

#### Case Grouping

Patients were divided into improvement and non-improvement groups based on whether neurological functioning improved in the early stages after thrombolytic therapy.

### Statistical Analysis

Statistical analysis was carried out using SAS 9.4 statistical software. Data that conformed to normal distributions were expressed by mean ± standard deviation (SD) and assessed using t-tests. A rank sum test was also used for comparison for data that did not conform to normal distributions. Enumeration data were represented by frequency and rate (%), and assessed by a *x*^2^ test. Multivariate Logistic stepwise regression analysis was performed for the factors with statistically significant differences in univariate analysis. The levels of blood glucose variability and inflammatory factors were compared between the two groups using Pearson linear correlation analysis. A *P* < 0.05 indicated a statistically significant difference.

## Results

### Univariate Analysis of Baseline Data

There were 63 patients in the improvement group and 83 patients in the non-improvement group, including 2 patients with symptomatic intracerebral hemorrhage. The proportion of diabetes and atrial fibrillation in the non-improvement group was higher than that in the improvement group (*P* < 0.05). The baseline NHISS score of the non-improvement group was higher than that of the improvement group, the non-improvement group had higher random blood glucose levels at admission than the improvement group, and glycosylated hemoglobin in the non-improvement group was significantly higher than in the improvement group (*P* < 0.05; Table 1).

**Table 1:** Baseline data for the improvement group and non-improvement group.

**Variable**	**Improvement group (*n* = 63)**	**Non-improvement group (*n* = 83)**	**t/Z/χ^2^**	***P***
Men (n, %)	38 (60.3%)	49 (59.0%)	0.024	0.876
Age (years, $\bar{x}$ ± s)	60.92 ± 11.88	64.10 ± 7.36	−1.868	0.065
Hypertension (n, %)	44 (69.8)	59 (71.1)	0.027	0.087
Diabetes (n, %)	15 (23.8)	34 (41.0)	4.727	0.030
Hyperlipidemia (n, %)	22 (34.9)	37 (44.6)	1.387	0.239
Coronary heart disease (n, %)	19 (30.2)	37 (44.6)	3.150	0.076
Atrial fibrillation (n, %)	11 (17.5)	33 (39.8)	8.458	0.004
Prior stroke/TIA (n, %)	16 (25.4)	19 (22.9)	0.123	0.725
Smoking history (n, %)	36 (57.1)	48 (57.8)	0.007	0.934
Drinking history (n, %)	23 (36.5)	31 (37.3)	0.011	0.917
Random blood glucose at admission (mmol/L, $\bar{x}$ ± s)	6.98 ± 1.30	7.51 ± 1.42	−2.331	0.021
Glycosylated hemoglobin (%, $\bar{x}$ ± s)	5.31 ± 1.00	6.37 ± 1.19	−5.835	0.000
Baseline NIHSS score ($\bar{x}$ ± s)	9.25 ± 3.67	11.01 ± 4.75	−2.525	0.013
OTT (min, $\bar{x}$ ± s)	169.37 ± 25.83	176.75 ± 23.74	−1.791	0.075

*NIHSS, National Institutes of Health Stroke Scale; OTT, onset to treatment*.

### Comparison of Blood Glucose Monitoring Indexes

The MBG, SDBG, MAGE and MVCBG levels of patients in the improvement group were significantly lower than those in the non-improvement group (*P* < 0.05; Table 2).

**Table 2:** Comparison of blood glucose monitoring indexes.

**Variable**	**Improvement group (n = 63)**	**Non-improvement group (n = 83)**	***t***	***P***
MBG (mmol/L, $\bar{x}$ ± s)	7.65 ± 1.25	8.21 ± 1.04	19.3226	<0.0001
SDBG (mmol/L, $\bar{x}$ ± s)	1.11 ± 0.41	1.65 ± 0.52	40.4752	<0.0001
MAGE (%, $\bar{x}$ ± s)	2.16 ± 0.86	3.06 ± 1.08	25.0631	<0.0001
MVCBG (%, $\bar{x}$ ± s)	14.32 ± 3.87	19.83 ± 4.55	42.9418	<0.0001

*MBG, mean blood glucose; SDBG, standard deviation of blood glucose; MAGE, mean amplitude of glycemic excursion; MVCBG, mean variation coefficient of blood glucose*.

### Comparison of Inflammatory Factor Levels

Patients in the improvement group had significantly lower levels of serum inflammatory factors, including IL-6, MMP-9, TNF-α and hs-CRP, than patients in the non-improvement group (*P* < 0.05; Table 3).

**Table 3:** Comparison of the levels of inflammatory factors.

**Variable**	**Improvement group (*n* = 63)**	**Non-improvement group (*n* = 83)**	***t***	***P***
TNF-α (ng/L, $\bar{x}$ ± s)	21.19 ± 4.11	28.45 ± 4.44	−10.099	0.000
IL-6 (μg/L, $\bar{x}$ ± s)	39.46 ± 2.53	44.33 ± 3.84	−9.229	0.000
MMP-9 (μg/L, $\bar{x}$ ± s)	117.75 ± 4.66	124.58 ± 4.45	−8.988	0.000
Hs-CRP (mg/L, $\bar{x}$ ± s)	4.25 ± 1.12	4.85 ± 1.26	−3.006	0.003

*TNF-α, tumor necrosis factor α; IL-6, interleukin-6; MMP-9, active matrix metalloproteinase-9; Hs-CRP, hypersensitive C-reactive protein*.

### Multivariate Logistic Regression Analysis

Variables with *P* < 0.05 in Tables 1, 2 (diabetes, atrial fibrillation, baseline NHISS scores, random blood glucose at admission, glycosylated hemoglobin, MBG, SDBG, MAGE, MVCBG) were selected for multivariate logistic regression analysis to analyze factors related to early symptom improvement. Results showed that baseline NIHSS scores, glycosylated hemoglobin, diabetes, MAGE, and MVCBG were significantly correlated with early symptom improvement after thrombolysis (Table 4).

**Table 4:** Multivariable analysis for early symptom improvement.

**Factors**	**B**	**SE**	**OR**	**95%CI**	***p***
Diabetes	2.57	1.11	13.10	(1.63, 131.45)	0.0210
Atrial fibrillation	−0.56	0.62	0.57	(0.16, 1.88)	0.3624
Random blood glucose	0.31	0.14	1.36	(1.05, 1.82)	0.7742
Glycosylated hemoglobin	0.94	0.20	2.57	(1.78, 3.98)	0.0005
Baseline NIHSS score	0.25	0.11	1.28	(1.05, 1.62)	0.0200
MBG	0.42	0.92	1.53	(0.21, 8.76)	0.6475
SDBG	−3.74	0.78	20.28	(7.05, 70.92)	0.9054
MAGE	−2.51	0.62	2.98	(1.92, 5.00)	<0.0001
MVCBG	−5.43	1.03	1.40	(1.26, 1.60)	0.0078

*NIHSS, National Institutes of Health Stroke Scale; MBG, mean blood glucose; SDBG, standard deviation of blood glucose; MAGE, mean amplitude of glycemic excursion; MVCBG, mean variation coefficient of blood glucose*.

### Correlation Analysis of Blood Glucose Variability Indices and Inflammatory Factors

Pearson linear correlation analysis showed that the blood glucose variability indices, including SDBG, MAGE, and MVCBG, were significantly positively correlated with the inflammatory factors IL-6, MMP-9, TNF-α and hs-CRP (*P* < 0.01; Table 5).

**Table 5:** Correlation analysis of blood glucose variability indices and inflammatory factors.

**Blood glucose variability**	**MMP-9**	**TNF-α**	**IL-6**	**CRP**
MVCBG	*r* = 0.527	*r* = 0.576	*r* = 0.590	*r* = 0.439
	*P* < 0.01	*P* < 0.01	*P* < 0.01	*P* < 0.01
MAGE	*r* = 0.579	*r* = 0.637	*r* = 0.640	*r* = 0.580
	*P* < 0.01	*P* < 0.01	*P* < 0.01	*P* < 0.01
SDBG	*r* = 0.623	*r* = 0.678	*r* = 0.682	*r* = 0.602
	*P* < 0.01	*P* < 0.01	*P* < 0.01	*P* < 0.01

*MVCBG, mean variation coefficient of blood glucose; MAGE, mean amplitude of glycemic excursion; SDBG, standard deviation of blood glucose; MMP-9, active matrix metalloproteinase-9; TNF-α, tumor necrosis factor α; IL-6, interleukin-6; Hs-CRP, hypersensitive C-reactive protein*.

## Discussion

Revascularization in patients with acute ischemic stroke has previously been associated with better outcomes. Early recovery of brain function has been considered to be an indicator of arterial recanalization and brain tissue reperfusion, and the relationship between vascular recanalization and early recovery of nerve function has been confirmed using multiple examination methods, such as transcranial Doppler imaging (2), CT angiography, magnetic resonance angiography, or digital subtraction angiography (DSA), etc. In this study, baseline NIHSS scores, glycosylated hemoglobin, diabetes, MAGE, and MVCBG were associated with early improvement after intravenous thrombolysis with alteplase in patients with AIS. High baseline NIHSS scores, high glycosylated hemoglobin levels, a history of diabetes, and high blood glucose variability were risk factors for decreased vascular recanalization.

At present, a number of studies have reported that hyperglycemia and diabetes are associated with the low rate of recanalization and poor clinical outcomes in IV rt-PA-treated patients (3). Therefore, how to control blood glucose more effectively, or if lowering blood glucose level alone is sufficient, has become a research hotspot (4). Blood glucose variability (blood glucose fluctuation) is also known as blood glucose drift and refers to the tendency of blood glucose levels to fluctuate. Between peaks and troughs, these changes are considered to be a third type of dysglycemia, in addition to hypoglycemia and hyperglycemia. Blood glucose variability can lead to vascular endothelial cell apoptosis via oxidative stress and inflammatory responses. Patients with acute cerebral infarctions are prone to stress hyperglycemia, so blood glucose fluctuations are more obvious (5). However, blood glucose variability has been consistently overlooked in stroke studies exploring the association between glycemia and clinical outcomes (6), and there are currently limited studies which have evaluated the association between blood glucose variability and clinical outcomes of stroke. There are also few studies that have investigated intravenous thrombolytic therapy related to stroke (7). A retrospective study assessed GV as the range of glucose (maximum-minimum) in diabetic AIS patients (8). It showed that high GV was associated with adverse functional outcomes, even after adjusting for hyperglycemic measures or hypoglycemic events. In one study, 336 diabetic AIS patients were recruited within 72 h of stroke onset, and the mean amplitude of glycemic excursion was found to be associated with early neurological deterioration (9). A study evaluating the association between GV indices and clinical outcomes in acute stroke patients showed that greater GV might be related to decreased odds of neurological improvement during hospitalization (10). Furthermore, a recent study demonstrated that high GV values during the first 48-h after ischaemic stroke were associated with increased mortality (11)_._ Patients treated with intravenous thrombolytic therapy and non-diabetic patients were included in this study. The results showed that the random blood glucose levels at admission, glycosylated hemoglobin, mean blood glucose levels and blood glucose variability indices (SDBG, MAGE, MVCBG) in the improvement group were lower than those in the non-improvement group. Differences in blood glucose variation, however, were more significant. We therefore suggest that blood glucose variability indices will better predict outcomes in AIS patients treated with intravenous alteplase thrombolysis, and that the sensitivity of MAGE and MVCBG is higher than that of SDBG.

The mechanism by which elevated blood glucose variability could lead to poor outcomes is not entirely clear. Some studies have confirmed that blood glucose fluctuation can induce the adhesion of inflammatory cytokines and vascular endothelial cells, aggravate body's inflammatory responses, and then affect the prognosis of patients with acute cerebral infarctions (12). Barbieri M et al. showed that both acute and chronic blood glucose fluctuations could induce inflammatory responses in T2DM patients. Compared with stable hyperglycemia, excessively large and frequent blood glucose fluctuations may cause more intense T2DM complications (13). Lowering the levels of blood glucose variability may reduce the levels of inflammatory factors, reduce the degree of neurological impairment in ACI patients, and improve their prognosis (14). TNF-α is one of the most important Th1 pro-inflammatory cytokines, and is considered a potential therapeutic target for acute stroke (15). As an important factor in the inflammatory response, Il-6 induces blood vessel damage during and following AIS (16). IL-6 can increase vascular endothelial permeability, improve coagulation factor activity, promote coagulation, and cause vascular dysfunction, which may ultimately lead to thrombosis and bleeding events (17). MMP-9 expression levels are low in normal brain tissue, but are increased after ischemic strokes. MMP-9 is involved in brain tissue injury through a number of mechanisms, including destruction of the blood-brain barrier (BBB), the promotion of inflammatory cell infiltration and inflammatory factor release, inducing nerve cell death and hemorrhage transformation of ischemic tissue (18, 19). C-reactive protein (CRP) is a sensitive acute reactive protein. Previous studies have shown that hs-CRP can predict the occurrence of ACI, and that the degree of neurological impairment in ACI patients is closely related to hs-CRP levels. Therefore, hs-CRP levels can indirectly indicate disease severity (20, 21).

Here, we selected four important inflammatory factors related to acute cerebral infarction as observation indicators, and found that their levels were significantly higher in the non-improvement group than in the improvement group. We therefore postulate that because IL-6, MMP-9, TNF-α and hs-CRP levels in AIS patients with high blood glucose variability who are treated with intravenous alteplase thrombolysis are significantly increased, the degree of inflammation is severe, which may lead to poor prognoses.

In summary, our study showed that blood glucose variability was significantly correlated with the early therapeutic effects of intravenous alteplase thrombolysis, as well as with the level of inflammatory factors in acute cerebral infarctions. If blood glucose fluctuates greatly, inflammatory reactions are obvious, and intravenous thrombolysis has a somewhat negligible effect. Thus, the reduction of blood glucose variability and inhibition of inflammatory responses has become a novel therapeutic mechanism for improving the prognosis of AIS patients. Clinicians should closely monitor and manage blood glucose variability in AIS patients. Blood glucose fluctuations should be controlled effectively, rather than by simply lowering blood glucose levels. Reducing blood glucose fluctuations may improve the prognosis of AIS patients treated with intravenous thrombolytic therapy.

Our study had some limitations. Since not all subjects were diabetic, fingernail blood glucose measurements were used to calculate blood glucose variability, rather than employing a continuous blood glucose monitoring method. In the future, we hope to apply a dynamic blood glucose monitoring system and obtain more accurate data. In addition, there is no widely accepted optimal index for blood glucose variability, leading to substantial reporting heterogeneity in relevant studies. Additionally, our sample size was small because this was a single-center study. A larger sample drawn from multiple centers would be useful for obtaining more reliable results in the future.

## Data Availability Statement

The original contributions presented in the study are included in the article/supplementary material, further inquiries can be directed to the corresponding author/s.

## Ethics Statement

The studies involving human participants were reviewed and approved by the Ethics Committee of Tianjin Huanhu Hospital. The patients/participants provided their written informed consent to participate in this study.

## Author Contributions

YC and PZ: study conception, design, analysis and interpretation of data, and critical revision of the manuscript for important intellectual content. YC and HZ: acquisition of data and drafting of the manuscript. YC, PZ, HZ, and QL: statistical analysis. All authors contributed to the article and approved the submitted version.

## Conflict of Interest

The authors declare that the research was conducted in the absence of any commercial or financial relationships that could be construed as a potential conflict of interest.

## Publisher's Note

All claims expressed in this article are solely those of the authors and do not necessarily represent those of their affiliated organizations, or those of the publisher, the editors and the reviewers. Any product that may be evaluated in this article, or claim that may be made by its manufacturer, is not guaranteed or endorsed by the publisher.

## References

[1] Powers WJ Rabinstein AA Ackerson T Adeoye OM Bambakidis NC Becker K. 2018 guidelines for the early management of patients with acute ischemic stroke: a guideline for healthcare professionals From the American Heart Association/American Stroke Association. Stroke. (2018) 49:e46–e110. 10.1161/STR.0000000000000158 29367334

[2] Eryildiz ES Özdemir AÖ. Factors associated with early recovery after intravenous thrombolytic therapy in acute ischemic stroke. Noro psikiyatri arsivi. (2018) 55:80–3. 10.29399/npa.22664 30042646 PMC6045809

[3] Saqqur M Shuaib A Alexandrov AV Sebastian J Khan K Uchino K. The correlation between admission blood glucose and intravenous rt-PA-induced arterial recanalization in acute ischemic stroke: a multi-centre TCD study. Int J Stroke. (2015) 10:1087–92. 10.1111/ijs.12517 26332252

[4] Arnold M Mattle S Galimanis A Kappeler L Fischer U Jung S. Impact of admission glucose and diabetes on recanalization and outcome after intra-arterial thrombolysis for ischaemic stroke. Int J Stroke. (2014) 9:985–91. 10.1111/j.1747-4949.2012.00879.x 22973986

[5] Sun Y Xu Y Geng L. Caspase-3 inhibitor prevents the apoptosis of brain tissue in rats with acute cerebral infarction. Exp Ther Med. (2015) 10:133–138. 10.3892/etm.2015.2462 26170924 PMC4487074

[6] Camara-Lemarroy CR. Glucose and stroke: what about glycemic variability. J Neurol Sci. (2017) 373:242–3. 10.1016/j.jns.2017.01.015 28131196

[7] Palaiodimou L Lioutas VA Lambadiari V Paraskevas GP Voumvourakis K Tsivgoulis G. Glycemia management in acute ischemic stroke: current concepts and novel therapeutic targets. Postgrad Med. (2019) 131:423–37. 10.1080/00325481.2019.1651206 31382796

[8] Kim YS Kim C Jung KH Kwon HM Heo SH Kim BJ. Range of glucose as a glycemic variability and 3-month outcome in diabetic patients with acute ischemic stroke. PLoS ONE. (2017) 12:e0183894. 10.1371/journal.pone.0183894 28880933 PMC5589173

[9] Hui J Zhang J Mao X Li Z Li X Wang F. The initial glycemic variability is associated with early neurological deterioration in diabetic patients with acute ischemic stroke. Neurol Sci. (2018) 39:1571–7. 10.1007/s10072-018-3463-6 29869743

[10] Palaiodimou L Lioutas VA Lambadiari V Theodorou A Themistocleous M Aponte L. Glycemic variability of acute stroke patients and clinical outcomes: a continuous glucose monitoring study. Ther Adv Neurol Disord. (2021) 14:17562864211045876. 10.1177/17562864211045876 eCollection 2021. 34589140 PMC8474316

[11] Gutiérrez-Zúñiga R Alonso de Leciñana M Delgado-Mederos R Gállego-Cullere J Rodríguez-Yáñez M Martínez-Zabaleta M. Beyond hyperglycemia: glycaemic variability as a prognostic factor after acute ischemic stroke. Neurologia. (2020) 2020:S0213-4853(20)30272-3. 10.1016/j.nrl.2020.06.018 37059570

[12] Hobbs J Tekes A Klein J Lemmon M Felling RJ Chavez-Valdez R. Acute paraplegia in a preterm infant with cerebral sinovenous thrombosis. J Perinatol. (2015) 35:460–2. 10.1038/jp.2015.26 26012477

[13] Chang C-M Hsieh C-J Huang J-C Huang I-C. Acute and chronic fluctuations in blood glucose levels can increase oxidative stress in type 2 diabetes mellitus. Acta Diabetol. (2012) 49 Suppl 1:S171–7. 10.1007/s00592-012-0398-x 22547264

[14] Patgiri D Pathak MS Sharma P Kutum T Mattack N. Serum hsCRP: a novel marker for prediction of cerebrovascular accidents (Stroke). J Clin Diagn Res. (2014) 8:CC08–11. 10.7860/JCDR/2014/10386.5302 25653940 PMC4316246

[15] Liu D-D Chu S-F Chen C Yang P-F Chen N-H He X. Research progress in stroke-induced immunodepression syndrome (SIDS) and stroke-associated pneumonia (SAP). Neurochem Int. (2018) 114:42–54. 10.1016/j.neuint.2018.01.002 29317279

[16] Croci D Nevzati E Danura H Schöpf S Fandino J Marbacher S. The relationship between IL-6, ET-1 and cerebral vasospasm, in experimental rabbit subarachnoid hemorrhage. J Neurosurg Sci. (2019) 63:245–50. 10.23736/S0390-5616.16.03876-5 27759738

[17] Gu Y Gutierrez J Meier IB Guzman VA Manly JJ Schupf N. Circulating inflammatory biomarkers are related to cerebrovascular disease in older adults. Neuroimmunol Neuroinflamm. (2019) 6:e521. 10.1212/NXI.0000000000000521, PMID:30568999 30568999 PMC6278856

[18] Li XF Zhang XJ Zhang C Wang LN Li YR Zhang Y. Ulinastatin protects brain against cerebral ischemia/reperfusion injury through inhibiting MMP-9 and alleviating loss of ZO-1 and occludin proteins in mice. Exp Neurol. (2018) 302:68–74. 10.1016/j.expneurol.2017.12.016 29291404

[19] Hannocks MJ Zhang X Gerwien H Chashchina A Burmeister M Korpos E. The gelatinases, MMP-2 and MMP-9, as fine tuners of neuroinflammatory processes. Matrix Biol. (2019) 75–76:102–13. 10.1016/j.matbio.2017.11.007 29158162

[20] de Sousa Parreira J Kallaur AP Lehmann MF Oliveira SR Alfieri DF Delongui F. Tumor necrosis factor beta NcoI polymorphism (rs909253) is associated with inflammatory and metabolic markers in acute ischemic stroke. Metab Brain Dis. (2015) 30:159–67. 10.1007/s11011-014-9584-6 25063351

[21] Das S Roy S Kaul S Jyothy A Munshi A CRP. gene (1059G>C) polymorphism and its plasma levels in ischemic stroke and hemorrhagic stroke in a south Indian population. Inflammation. (2014) 37:1683–88. 10.1007/s10753-014-9897-y 24771073

